# Evolvability of feed-forward loop architecture biases its abundance in transcription networks

**DOI:** 10.1186/1752-0509-6-7

**Published:** 2012-01-19

**Authors:** Stefanie Widder, Ricard Solé, Javier Macía

**Affiliations:** 1 Department for Computational Systems Biology, University of Vienna, Althanstr. 14, A-1090 Vienna, Austria; 2 Complex Systems Lab (ICREA-UPF), Barcelona Biomedical Research Park (PRBB-GRIB), Dr. Aiguader 88, 08003 Barcelona, Spain; 3 Santa Fe Institute, 1399 Hyde Park Road, Santa Fe NM87501, USA

## Abstract

**Background:**

Transcription networks define the core of the regulatory machinery of cellular life and are largely responsible for information processing and decision making. At the small scale, interaction motifs have been characterized based on their abundance and some seemingly general patterns have been described. In particular, the abundance of different feed-forward loop motifs in gene regulatory networks displays systematic biases towards some particular topologies, which are much more common than others. The causative process of this pattern is still matter of debate.

**Results:**

We analyzed the entire motif-function landscape of the feed-forward loop using the formalism developed in a previous work. We evaluated the probabilities to implement possible functions for each motif and found that the kurtosis of these distributions correlate well with the natural abundance pattern. Kurtosis is a standard measure for the peakedness of probability distributions. Furthermore, we examined the functional robustness of the motifs facing mutational pressure *in silico *and observed that the abundance pattern is biased by the degree of their evolvability.

**Conclusions:**

The natural abundance pattern of the feed-forward loop can be reconstructed concerning its intrinsic plasticity. Intrinsic plasticity is associated to each motif in terms of its capacity of implementing a repertoire of possible functions and it is directly linked to the motif's evolvability. Since evolvability is defined as the potential phenotypic variation of the motif upon mutation, the link plausibly explains the abundance pattern.

## Background

Evolutionary adaptability in biological systems is often the result of trade-offs between flexibility and specialization [1]. In this context, buffering mutations and noise seem an important requirement for stability. This can be achieved by a robust response to parameter changes and correlates with the degree of specialization of the given structure. A given network insensitive to mutations will always perform the same function. On the other hand, adaptation and evolvability requires flexible structures that can be re-used to perform different (potential) functions and thus provide plasticity [2,3]. The problem here is often understanding why some particular structures are so common and what their (if any) functional meaning is. This is closely tied to the mapping *f *between structure *S *and function *F*, namely the relationship:

(1) $$S\overset{f}{\to }F$$

which is usually dubbed as the *genotype-phenotype *mapping problem [4]. Understanding the nature and origins of this mapping is at the core of many key questions concerning the evolution of complexity in nature.

Within the context of gene transcription networks, it has been suggested that the previous problem can be dissected by analyzing the frequency of some overabundant sub-networks of three or four elements, so called *network motifs *[5-7]. These sub-graphs only capture the topological pattern of connections and a dynamical description of their potential function requires a set of differential equations [8,9]. One particularly important example is provided by feed-forward loop (FFL) motifs [5]. Many genetic and biochemical systems, such as the Lac and Che systems in *E. coli *(responsible for lactose utilization and chemotaxis, respectively) involve FFL motifs [10-12]. Mounting evidence indicates that they have key roles in cell function [10] and morphogenesis [13,14].

However, the origin of a preferential bias towards given topologies remains under discussion.

**Figure 1:**
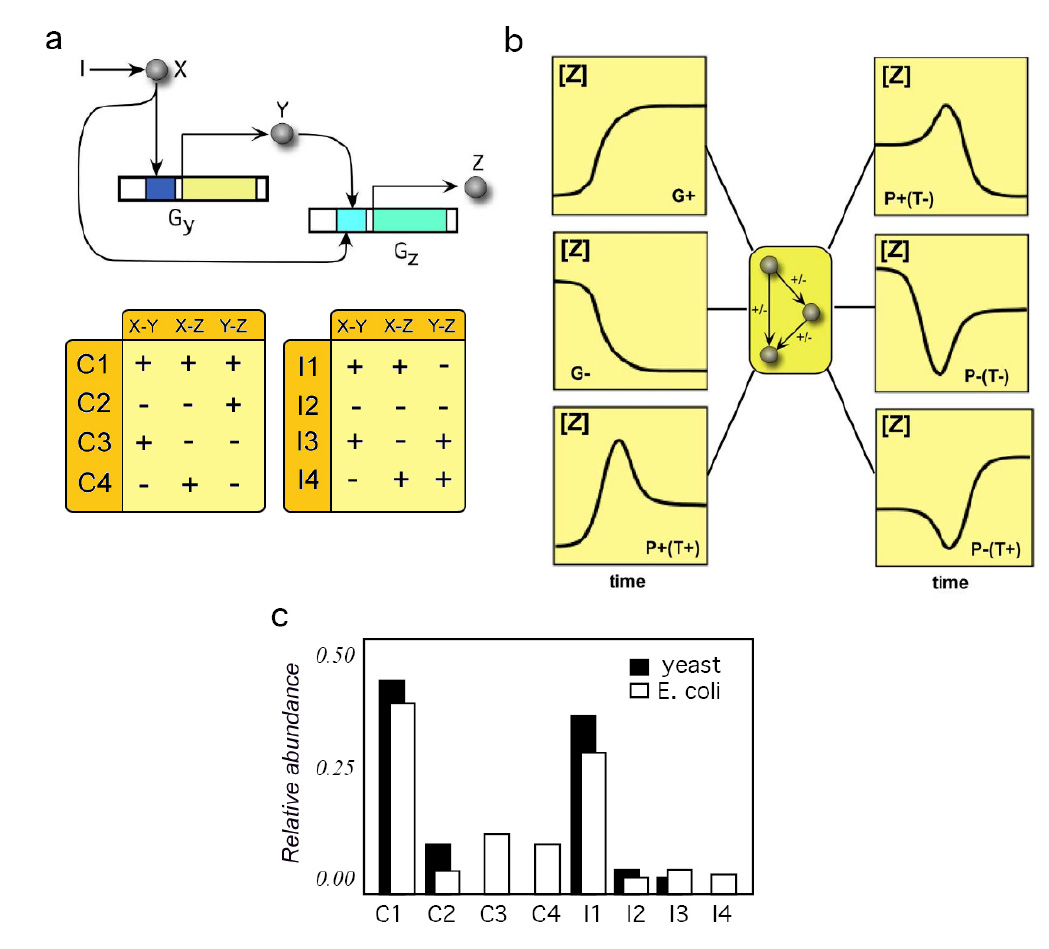
**Structure and frequency of FFL motifs**. In (a) we show the schematic representation of the FFL's genetic regulatory interactions ('+' represents activatory regulation and '-' represents inhibitory regulation). The external input *I *activates the signal protein *X*. Active *X *modulates expression of gene *G*_*Z *_directly and indirectly via regulation of *Y *expression, which in turn also regulates *G*_*Z*_. The dynamics of these regulatory interactions is described by a set of equations *dy/dt *= *F*(*y, z*), *dz/dt *= *G*(*y, z*) incorporating the nonlinearities associated to gene-gene interactions. In (b) we plot the general topology of FFL motifs and the six different functions *ϕ*(*t*) represented by qualitative time-courses [*Z*(*t*)]. 'G' indicates grader dynamics, 'P' pulser dynamics. We specifically take into account the initial slope of the time-course ('+' or '-') and the concentration of the final target *Z *with respect to the non-induced protein concentration ('T+' and 'T-'). In (c) we display the relative abundance *P*_*obs*_(Γ_*i*_) of these motifs in the transcription networks of yeast and *E. coli *(data from [27]).

The relative frequency of FFLs displays a well-defined pattern (figure 1c) dominated by two sub-graphs (C1 and I1). The uneven abundance of these graphs could be a fingerprint of their functional relevance [8,15-17]. Such importance would be the blueprint of an evolutionary advantage, but it is not clear whether such functional connection really exists [15,18-21] or if it resembles instead a byproduct of non-adaptive processes [22-25]. As shown below, motif structure does not directly relate to its frequency, but its plasticity in implementing different functions does.

## Results and Discussion

### Probability distribution of implementing different functions

Consider the FFL graphs Γ_*i *_from the set $S=\left\{C_{1},C_{2},C_{3},C_{4},I_{1},I_{2},I_{3},I_{4}\right\}$ shown in figure 1a. In previous studies [9,26] it has been shown that, the topology of a given FFL does not univocally define its function but it captures the probability distribution of implementing *different *functions. Our first goal is to identify an appropriate mapping *f *between FFL topology and each potential response *ϕ*_*j*_(*t*), i.e. *f*_*ij *_: {Γ_*i*_, *x*(0), *μ*} → *ϕ*_*j*_(*t*) where we indicate as {Γ_*i*_, *x*(0),*μ*} the FFL graph together with its initial condition *x*(0) and the set of parameters used *μ *[26]. The six different responses (figure 1b) are triggered by an external input. These are either fast response (pulser) or delayed response (grader) considering the target concentration of the output. Here *f*_*ij *_indicates the likelihood that *ϕ*_*j*_(*t*) is implemented by Γ_*i*_.

**Figure 2:**
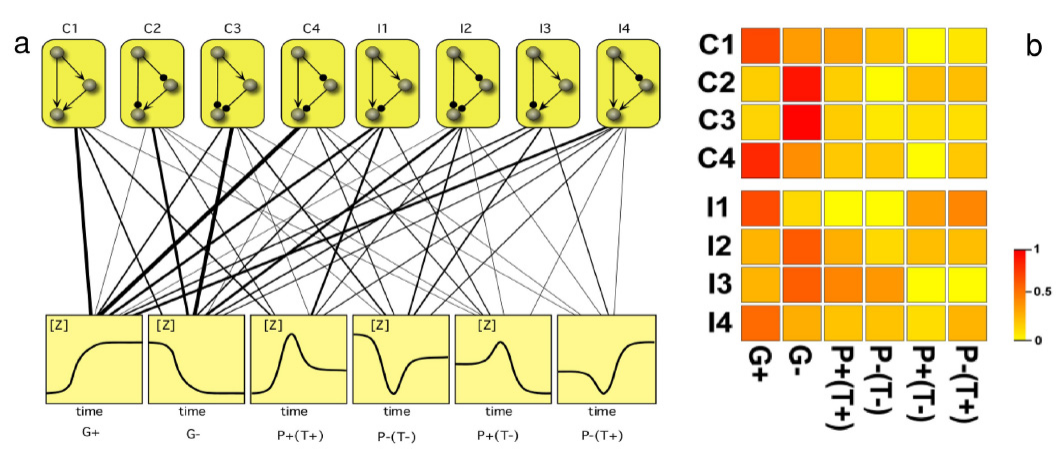
**FFL function and their probabilities**. (a) The landscape of FFL motifs is displayed as a bipartite graph linking patterns (upper row) and processes (lower). The weight of the links indicates the relative probability *P*(*ϕ*_*j *_| Γ_*i*_) that a given motif Γ_*i *_implements a given function *ϕ*_*j*_. In (b) the matrix of motif-function probabilities is displayed using a color scale. The plots highlight that some motifs look more specialized, whereas others display rather evenly distributed functional responses.

How likely is a motif to become part of a complex cellular network? Two extreme strategies can be envisioned. In the first, specific motifs play specific roles in a robust way and they are common because they are insensitive to mutational noise. In the second, the larger the variety of implementable functions, the more flexible the better. Such a scenario is feasible under the premise of ever-changing environments and comes with the cost of reduced robustness. In order to measure the plasticity of decision-making between these two strategies, let us first determine the (conditional) probability *f*_*ij *_= *P*(*ϕ*_*j*_|Γ_*i*_). These probabilities are normalized, i. e. $\sum_{\left\{\phi_{j}\right\}}P\left(\phi_{j}|\Gamma_{i}\right)=1$ and can be systematically computed [26]. This set actually defines our structure-function map, namely

(2) $$\Gamma_{i}\overset{f_{ij}}{\to }\phi_{j}$$

and can be displayed (figure 2a) as a weighted, motif-function bipartite graph (see Methods). The graph reveals that most motifs implement all functions, but the likelihood of each pair is case-dependent. Some motifs seem clearly more specialized (such as *C*4) whereas others are rather generalists (see for example *I*4). What influences the choice of a given topology over others? Since most of the functions can be implemented, it is not clear that a one-to-one, function-based argument will work. But we can go a step beyond and look at the structure of the probability distribution {*P*(*ϕ*_*j*_|Γ_*i*_)} of each topology. This can be done by measuring the degree of homogeneity displayed, for each Γ_*i*_, by plotting *P*(*ϕ*_*j*_|Γ_*i*_). If the motif is highly specialized, some dominant peak(s) would be observed, whereas if it is very flexible no prominent peaks will appear. A simple way of measuring the homogeneity of the distribution is given by its kurtosis, defined as the fourth standardized moment by

(3) $$K=\frac{k_{4}}{k_{2}^{2}}-3$$

Kurtosis is the measure of the "peakedness" of a distribution. It quantifies the concentration of frequencies around the mean of the distribution. Higher kurtosis means that the variance is the result of infrequent, extreme deviations from the mean as opposed to frequent, modestly sized deviations resulting in low kurtosis. In order to define a measure characterizing the degree of plasticity of a given motif in terms of its specialization or its flexibility, we can consider two extreme cases, namely the most flexible graph Γ_*f *_equally likely to implement any function *ϕ*_*j*_, and the most specialized graph Γ_*s *_implementing only one function *ϕ*_1_. In the first case we would have *P*(*ϕ*_*j *_*|Γ*_*f*_) = 1/6 and the kurtosis associated is *K*(*Γ*_*f*_) = -3.33, whereas in the second case we would have *P*(*Γ*_*s*_) = 1 and 0 otherwise, with kurtosis *K*(*Γ*_*s*_) = 6. Details on the calculation can be found in Methods. Any other FFL graph Γ_*i *_from the set $S=\left\{C_{1},C_{2},C_{3},C_{4},I_{1},I_{2},I_{3},I_{4}\right\}$ has kurtosis values locating within this interval, *K*(Γ_*i*_) ∈ (*K*(Γ_*f*_), *K*(Γ_*s*_)).

In order to measure the degree of plasticity in the decision-making between these extreme cases we introduce *ψ*(Γ_*i*_)*. ψ*(Γ_*i*_) is the distance between the absolute value of the kurtosis *K*(Γ_*i*_) and the origin *K*_0 _[26] or in other words represents the intermediate level between specialization and flexibility. This transformation opens the way for a more intuitive biological interpretation: The values for *ψ*(Γ_*i*_) range between high plasticity (low *ψ*(Γ_*i*_)) and high commitment towards one of the extreme strategies, i.e. maximal specialization or maximal flexibility (high *ψ*(Γ_*i*_)). The optimal solution here is likely to be strongly impacted by the predictability of the environment. As a first approximation we therefor place *K*_0 _at the midpoint of the interval (*K*_*f*_,*K*_*s*_), i.e.

(4) $$\psi \left(\Gamma_{i}\right)=|K\left(\Gamma_{i}\right)|-K_{0}$$

Finally we define the likelihood *ρ*(Γ_*i*_) of a given motif Γ_*i *_to appear within a network as a function of its *ψ*(Γ_*i*_). Assuming that a high degree of flexibility or specialization should be related with lower likelihood of appearance for a given motif Γ_*i*_, we write

(5) $$\rho \left(\Gamma_{i}\right)=\frac{1}{\alpha }\psi {\left(\Gamma_{i}\right)}^{-1}$$

where *α *is a normalization coefficient defined as $\alpha =\sum_{j=1}^{8}{\psi \left(\Gamma_{j}\right)}^{-1}$.

**Figure 3:**
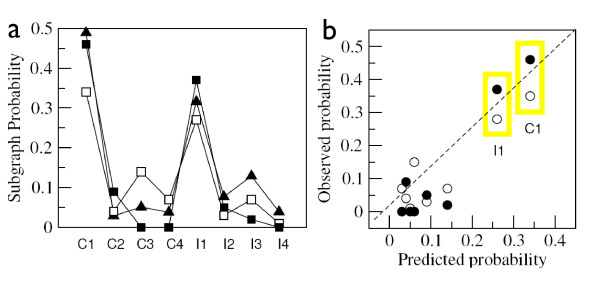
**Predicted probability and FFL abundance**. In (a) we compare the natural abundance and its predicted counterpart *ρ*(Γ_*i*_)*. S. cerevisiae *(black box) is compared to *E. coli *(white box) and the predicted probabilities (black triangle). In (b) we present the correlation between *ρ*(Γ_*i*_) and the natural abundances. The Pearson coefficient for the linear fit is *r *= 0.91 and *r *= 0.94 for *E. coli *and *S. cerevisiae*, respectively.

This function actually defines the expected probability of finding a given sub-graph and is thus mapping between the distributions associated to each motif and the expected abundance of motifs within networks. Figures 3a and 3b show the correlation between relative abundances of FFL motifs in *E. coli *and *S. cerevisiae *with respect to their expected probabilities *ρ*(Γ_*i*_). The matching is striking (data concerning abundances obtained from [27]). The two most abundant graphs (C1 and I1) are consistent with our results and the actual distribution matches well the observed pattern.

**Figure 4:**
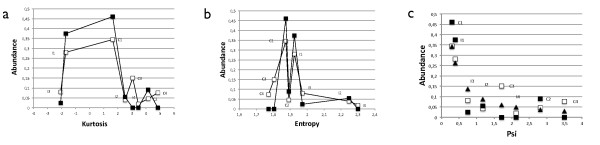
**FFL plasticity and abundance**. Here we compare the measures kurtosis, entropy and ψ(Γ_*i*_) and their correlation with the abundance of NW motifs (S. *cerevisiae *shown in black and *E.coli *in white). In (a) the kurtosis of the motif's probability distribution for different functions versus the motifs abundance is plotted. In (b) we show entropy versus abundance. The most abundant motifs have intermediate values for both measures which can be interpreted as high plasticity in both cases. In (c) we present the correlation between *ψ*(Γ_*i*_) and abundance. *ψ*(*γ*_*i*_) correlates negatively and thus again, plasticity correlates positively with motif abundance.

Interestingly, the expected probabilities indicate a positive bias toward systems which show high plasticity as presented in figure 4. Intermediate values for kurtosis (figure 4a) or low *ψ*(Γ_*i*_) (figure 4c) correlate with an increase in the likelihood of appearance. The values for kurtosis, *ψ*(Γ_*i*_) and *ρ*(Γ_*i*_) are collected in table 1. As an alternative measure for the homogeneity of a probability distribution the Shannon entropy is discussed in the Methods section, where the motif's entropy is used to characterize the degree of flexibility or specialization of a given motif Γ_*i *_[26]. Both, entropy (figure 4b) and kurtosis (figure 4a) yield similar results, ranking the most abundant motifs with intermediate values which translates to high functional plasticity.

**Table 1:** Kurtosis, *ψ*(Γ_*i*_) and predicted probability *ρ*(Γ_*i*_)

	*Kurtosis*	***ψ*(Γ**_***i***_**)**	***ρ*(Γ**_***i***_**)**
*C*1	1.631	0.297	0.342
*C*2	4.142	2.808	0.036
*C*3	3.042	1.707	0.059
*C*4	4.835	3.501	0.029
*I*1	-1.721	0.386	0.263
*I*2	2.506	1.17	0.087
I3	-2.083	0.748	0.136
*I*4	3.459	2.123	0.048

Table 1 shows the values for the motifs' kurtosis, *ψ*(Γ_*i*_) and predicted probability *ρ*(*Γi*). The entries are calculated from the probability distributions according to equations 3,4 and 7.

For the less abundant motifs we see a more disordered trend in the two measures, as is the case for C3, C4, I3 (both measures) or C2 (entropy). The interpretation here is not straightforward. It is feasible that the disordered trends can be consequence of non-adaptive processes. An alternative hypothesis is related to the shape of the *real *distributions for the implementation of any function. We assume that for more and less frequent motifs the analytically deduced probability distributions does not fit equally well the real counterpart. The more abundant the motif, the better the underlying probability distribution is mirrored in its abundance, because the sampling space is covered more readily.

Our analysis of FFLs dynamics was performed considering single, isolated motifs. However, in real systems motifs are embedded in large networks allowing for the combination of motifs. The combination of more abundant motifs, such as C1 and I1, can cover the whole set of possible dynamics by that affecting the abundance of the rest of the motifs.

### Evolvability

**Figure 5:**
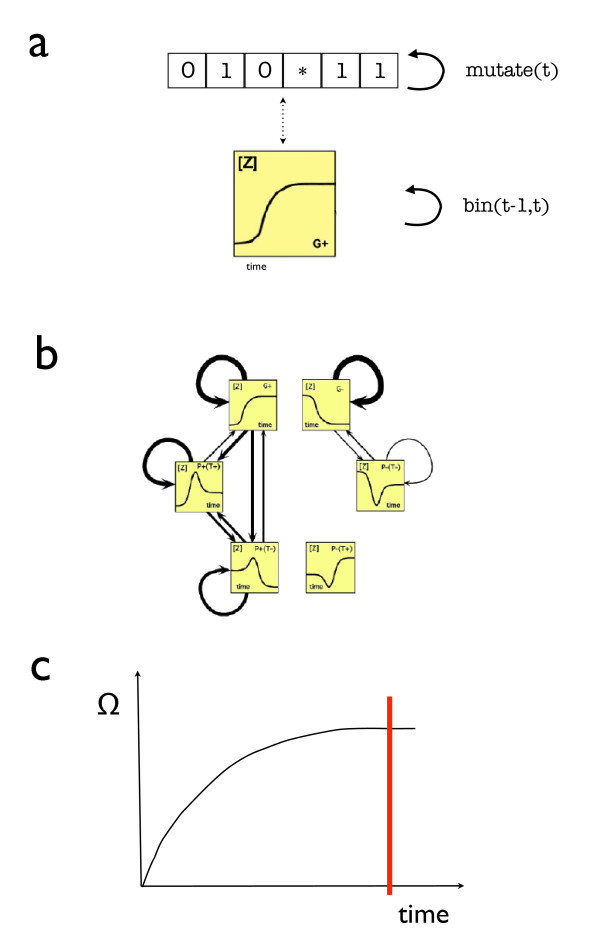
**Computation of the transition frequencies**. Sketch of the procedure. In (a) the update rule is shown. For a given string of conditional relations (BR) the associated dynamical pattern is calculated at time-step *t - *1. Next the entries of BR are mutated at time-step *t *and the (new) dynamical pattern is evaluated. Then the transition of pattern_*t*-1 _to pattern_*t *_is binned. This protocol is executed until no more changes in the bins occur as shown in plot (c). In (b) the graph Ω_*m*_(Γ_*i*_) associated to the transitions between the possible types of dynamics is represented for *C*1. The thickness of the arrows correspond to transition probabilities obtained from procedure (a). From these graphs (see Methods) we can calculate the motif evolvability ***ε***, which is found to positively correlate with *ρ*(Γ_*i*_).

In order to have a relevant role in evolvability, the degree of plasticity of FFLs should correlate with the motif's capacity of generating phenotypic variation by exploring different functions under mutation. Two key aspects are of importance here, namely i) the reduction of mutational lethality and ii) the up-speeding of adaptational processes (reduction of the number of mutations needed to generate new phenotypes [1]). The evolvability of the circuits Γ_*k *_can be studied by calculating the transition probabilities $\omega_{k}^{m}\left(\phi_{j}|\phi_{i}\right)$ of shifting from function *ϕ*_*i *_to *ϕ*_*j *_under *m *mutations. The matrix $\Omega_{m}\left(k\right)=\left(\omega_{k}^{m}\right)$ defines a flow graph (figure 5b) which allows us quantifying its evolvability $\varepsilon \left(\Gamma_{i}\right)$. We compared the robustness against single mutations versus sequential accumulation of multiple mutations. Mutations *m *are defined as single parameter changes. For better understanding of the procedure, we want to stress the conceptual difference of continuous *real *mutations and the here applied parameter changes *m *which result in a discretized observation pattern. In the presented framework a mutation *m *can be both, numerically small or large without impact as long as it does not drive the system into another functional regime.

**Figure 6:**
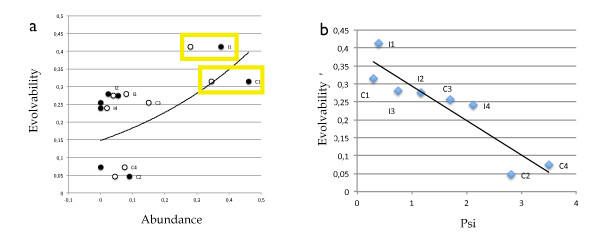
**FFL evolvability**. In plot (a) we show the correlation between the motif's evolvability and its abundance. Evolvability ***ε***, which is found to positively correlate with *ρ*(Γ_*i*_), is highest for the most abundant motifs *C*1*, I*1. In black we show data point of *S. cerevisiae*, in white *E. coli*. In (b) we show the correlation between the FFL's evolvability and its *ψ*(Γ_*i*_). We calculate a Pearson coefficient of *r *= -0.92 for the linear fit. The lower *ψ*(Γ_*i*_), the higher the motif's plasticity and the higher its evolvability. The data points are developed from the motifs topology and thus are species independent (blue).

Robustness is defined as the sum of the diagonal elements of Ω_*m*_(*k*), i. e. $\sum_{i}\omega_{k}^{m}\left(\phi_{i}|\phi_{i}\right)$. The diagonal elements $\omega_{k}^{m}\left(\phi_{i}|\phi_{i}\right)$ give the probabilities of performing the same function after *m *mutations. A detailed description of the procedure is given in Methods. We have found that FFL motifs are very robust but some of them exhibit a high phenotypic variation under repeated mutations. The most abundant motifs, *C*1 and *I*1, show the highest phenotypic variation. In other words, *C*1 and *I*1 can widely change their function with greater ease than the rest of the circuits facilitated by their low *ψ*(Γ_*i*_) (figure 6b). A network displaying little phenotypic diversity would give small values of ***ε*** whereas sub-graphs with high transition rates among states will have a high ***ε***. As presented in figure 6a, ***ε*** correlates positively with the abundance of motifs, with C1 and I1 displaying the largest values. These results suggest that a proper degree of plasticity, in terms of a balance between flexibility and specialization, is the optimal strategy to increase evolvability providing the playground for adaptive responses without increasing mutational lethality.

Assuming that motif plasticity is a relevant trait, our analysis supports the idea that the observed FFL abundance pattern actually correlates with motif evolvability.

Our analysis suggests that neither a direct interpretation of motifs as functional modules [1,2,4] nor a purely non-adaptive view of their abundance [22-24] account for the uneven presence in transcription networks. Consistently with previous works [28,29] duplication-rewiring dynamics alone cannot explain the evolution of FFLs. The potential for evolvability associated to their topological structure might well be the missing ingredient connecting both views.

## Conclusions

In this article we have interpreted a simplified, qualitative model of the FFL motif. The thorough analysis within the model framework allows to reconstruct its natural abundance pattern and provides insight in what might have shaped it. The argument leads to the very core of the *genotype-phenotype *mapping problem, since, due to its simplicity, a perfect mapping between the topology and all possible functions it can implement can be constituted. We claim, however, general applicability. FFL abundances are correlated with their plasticity and evolvability. Evolvability has been defined as a compromise between robustness against single mutations and the capability to modify the functional response upon increasing mutational pressure. The results indicate that a proper portion of intrinsic functional plasticity, which can be understood as a strategic trade-off between specialization and flexibility, is necessary to be abundant. Because only then one is suited to be readily evolvable in changing environments.

Future work should be devoted to analyzing how the coexistence of different motifs embedded in a large network affects their dynamics and abundance compared to the single motif analysis performed in this work.

## Methods

### Dynamical response of FFL motifs

Network motifs are recurrent interaction patterns, which are significantly more often encountered in biological interaction graphs than expected from random nets. It has been shown that feed forward loops (FFL) are capable of processing external signals by responding in a very specific, robust manner, either accelerating or delaying responses. They are composed of three genes. Firstly, gene *G*_*X *_that expresses the protein *X*. This protein *X *regulates the expression of the other two genes *G*_*Y *_and *G*_*Z *_encoding the proteins *Y *and *Z*, respectively. Additionally, protein *Y *regulates the expression of *Z *(see figure 1). Here, we assume that expression of *X *is unregulated and the protein is expressed in its inactive form, i.e. *X *does not regulate the expressions of *Y *and *Z *straightaway. Only upon the presence of an external signal (the input) *X *becomes active and regulation of *Y *and *Z *takes place, where *Z *resembles the output of the motif. The dynamics describing how the concentration of *Z *changes during time from the initial state (without input) to the final state (with input) are calculated from a set of two differential equations [26]:

(6) $$\begin{array}{c} \;\;\;\;\;\;\;\;\;\;Ẏ=\gamma_{Y}\left(\frac{1+\alpha^{x}\omega_{y}^{x}X^{n}}{1+\omega_{y}^{x}X^{n}}\right)-\delta_{y}Y\\ Ż=\gamma_{Z}\left(\frac{1+\beta^{x}\omega_{z}^{x}X^{n}+\beta^{y}\omega_{z}^{y}Y^{m}+\beta^{xy}\omega_{z}^{xy}X^{n}Y^{m}}{1+\omega_{z}^{x}X^{n}+\omega_{z}^{y}Y^{m}+\omega_{z}^{xy}X^{n}Y^{m}}\right)-\delta_{z}Z \end{array}$$

Here γ_*i *_describes the basal production of protein *i*, with *i *= {*Y, Z*}, subsuming the concentration of all biochemical elements which remain constant in time. The binding equilibrium of the regulators *j *with the gene *G*_*i *_are denoted by $\omega_{i}^{j}$, with *j *= {*X, Y, Z*}. Parameters α^*x *^and *β*^*j *^define the type of regulatory interactions, i.e. activation or inhibition, for gene *G*_*Y *_and *G*_*Z*_, respectively, providing the regulatory rates with respect to the basal transcription. Values < 1 correspond to inhibitory regulation, whereas > 1 accounts for activation (denoted by '-' and '+' in figure 1a respectively). The parameter *β*^*xy *^accounts for the simultaneous regulation of *G*_*Z*_. The degradation rate of protein *i *is denoted as *δ*_*i*_. Finally, *n *and *m *are the degree of multimerization of the regulators.

If we consider the system in phase space, we find that in absence of input the system resides in a stable steady state determined by the crossing of the nullclines, *Ẏ *= 0 and *Ż *= 0, respectively. Upon external input, *X *is activated and hence the shapes of the nullclines change. They provide a new crossing and consequently a new steady state. Due to these changes in the nullclines' geometry the system must evolve from the initial state towards the new stable state. The evolution corresponds to a trajectory crossing phase space that depends on i) the location of the initial state, ii) the location of the final state, iii) the new shapes of the nullclines upon input. The specific dynamics implemented by a given motif is determined by this trajectory, which depends on the set of parameters. However, by analyzing the geometrical features of the nullclines it is possible to determine the so-called *Backbone of Requirements for the FFL response *(BR), i.e. a set of qualitative relationships between different geometrical features of the nullclines and the location of the initial and the final point that univocally determines the dynamics [26]. Therefore, for a given FFL motif all different sets of parameters satisfying the same BR implement the same function. Similarly, also different BRs may implement the same function.

Based on the analysis of the different BRs associated with a given motif and their impact on the motif's function, we are in the position to determine a distribution of probabilities for the implementation of any function (see [26] for details about quantification of the dynamical probabilities). Table 2 shows the conditional probability that each motif Γ_*i *_(rows) implements the function *ϕ*_*j *_(columns).

**Table 2:** Conditional probabilities *P*(*ϕ*_*j *_| Γ_*i*_)

	***G***^**+**^	***G***^-^	***P***^**+**^***T***^**+**^	***P***^**+**^***T ***^**-**^	***P***^**-**^***T***^** -**^	***P***^**-**^***T***^**+**^
*C*1	0.4862	0.2111	0.2018	0.03669	0.0642	0
*C*2	0.0931	0.5349	0.1861	0.09302	0.0931	0
*C*3	0.2336	0.5514	0.0748	0.09346	0.0374	0.0093
*C*4	0.5862	0.0689	0.1379	0.13793	0	0.0689
*I*1	0.3571	0.2143	0.2857	0	0.1429	0
*I*2	0.1111	0.4167	0.1111	0.2222	0.0648	0.0741
*I*3	0.2553	0.2766	0	0.2979	0	0.1702
*I*4	0.4019	0.1402	0.1869	0.1308	0.0748	0.0654

Table 2 shows the conditional probability for each motif Γ_*i *_(rows) to implement the function *ϕ*_*j *_(columns).These probability distributions are calculated from the association of a given motif, its possible BRs and the respective functions, as described in [26].

### Functional robustness and mutational perturbation

Parametric mutations have different impact on the motif's function, as in nature they can either be neutral or causing qualitative changes. For the system we present here, only those mutations cause functional change, which induce a qualitative alteration in the shape of the nullclines, represented in the *Backbone of requirements for the FFL response *(BR) [26]. However, the mutation will become visible only, if the resulting BR is actually associated with a different function.

To estimate and compare the degree of mutational robustness for the different FFL motifs, we carried out a numerical study calculating the frequency of functional shifts upon parametric perturbation of equation (6) as shown in figure 5. For a given motif, this can be done introducing random mutations in the parameters that define characteristically the dynamics of FFL motif (figure 5a). We restrict the analysis to that sort of mutations that does not change the topology of the FFL, i.e. mutations that do not change the qualitative type of regulations (activation or inhibition) described by *a*^*x*^, *β*^*x *^and *β*^*y*^.

The suffered mutations are reflected (or not) in a qualitative change of the BR. Here different scenarios are possible, i) mutations that do not affect qualitatively the nullclines' geometry, i.e. there are no changes in the BR (neutral), ii) mutations that are reflected in compatible changes in the BR, but the new BR is associated to the same dynamic than the previous one (neutral), and finally iii) mutations that are reflected in compatible changes in the BR, and the new BR is associated to a different dynamic (qualitatively changing mutations).

In our numerical study we have considered 1000 different sets of parameters for each FFL type (8000 circuits in total). For each FFL, the mutational process is repeated 10.000 times until the probabilities of functional shifts stabilize (figure 5c) and the effects of the accumulation of mutations can be analyzed. This evaluation of the transition probabilities does not depend on specific parameter values but on the conditional relations between them. Since the relations are of the sort *a *> 6, changes in the function may be achieved by very small or large parameter changes equally likely, depending only on the conditional dependencies between the key-parameters and the corresponding values at time-step *t *- 1.

Tables 3 and 4 show the probabilities of transition between the different dynamics subsumed in a transition matrix. These matrices define a transition graph for each FFL motif shown in figure 5b.

**Table 3:** Transition probabilities for single mutations C1-C4

	***G***^**+**^	***G***^-^	***P***^**+**^***T***^**+**^	***P***^-^***T***^-^	***P***^**+**^***T***^-^	***P***^-^***T***^**+**^
**C1**						

*G*^+^	0.251	0	0.008	0	0	0
*G*^-^	0	0.329	0	0	0.003	0
*P*^+^*T*^+^	0.015	0	0.180	0.013	0	0
*P*^-^*T*^-^	0	0	0.015	0.157	0	0
*P*^+^*T*^-^	0	0.0049	0	0	0.031	0
*P*^-^*T*^+^	0	0	0	0	0	0

**C2**						

*G*^+^	0	0	0.007	0	0	0
*G*^-^	0	0.82	0	0	0.007	0
*P*^+^*T*^+^	0.008	0	0.022	0.001	0	0
*P*^-^*T*^-^	0	0	0.043	0.039	0	0
*P*^+^*T*^-^	0	0.007	0	0	0.039	0
*P*^-^*T*^+^	0	0	0	0	0	0

**C3**						

*G*^+^	0.317	0	0.008	0	0	0
*G*^-^	0	0.242	0	0	0.026	0
*P*^+^*T*^+^	0.023	0	0.030	0	0	0
*P*^-^*T*^-^	0	0	0	0	0	0
*P*^+^*T*^-^	0	0.043	0	0	0.174	0.046
*P*^-^*T*^+^	0	0	0	0	0.046	0.151

**C4**						

*G*^+^	0.815	0	0.019	0	0	0
*G*^-^	0	0	0	0	0.019	0
*P*^+^*T*^+^	0	0	0.037	0	0	0
*P*^-^*T*^-^	0	0	0	0	0	0
*P*^+^*T*^-^	0	0.037	0	0	0.037	0
*P*^-^*T*^+^	0	0	0	0	0	0.037

Table 3,4,5 and 6 list the transition probabilities between functions for every motif and its respective function probability distribution. The values are obtained from numerical simulation.

**Table 4:** Transition probabilities for single mutations I1-I4

	***G***^**+**^	***G***^**-**^	***P***^**+**^***T***^**+**^	***P***^**-**^***T***^**-**^	***P***^**+**^***T***^**-**^	***P***^**-**^***T***^**+**^
**I1**						

*G*^+^	0.241	0	0.069	0	0	0
*G*^-^	0	0.103	0	0	0	0
*P*^+^*T*^+^	0	0	0.345	0	0	0
*P*^-^*T*^-^	0	0	0	0.241	0	0
*P*^+^*T*^-^	0	0	0	0	0	0
*P*^-^*T*^+^	0	0	0	0	0	0

**I2**						

*G*^+^	0.144	0	0.024	0	0	0
*G*^-^	0	0.408	0	0	0.016	0
*P*^+^*T*^+^	0	0	0.160	0	0	0
*P*^-^*T*^-^	0	0	0	0.128	0	0
*P*^+^*T*^-^	0	0.032	0	0	0.080	0
*P*^-^*T*^+^	0	0	0	0	0	0.008

**I3**						

*G*^+^	0.091	0	0	0	0	0
*G*^-^	0	0.212	0	0	0.061	0
*P*^+^*T*^+^	0	0	0	0	0	0
*P*^-^*T*^-^	0	0	0	0	0	0
*P*^+^*T*^-^	0	0.121	0	0	0.394	0
*P*^-^*T*^+^	0	0	0	0	0	0.121

**I4**						

*G*^+^	0.398	0	0.008	0	0	0
*G*^-^	0	0.141	0	0	0.023	0
*P*^+^*T*^+^	0	0	0.063	0	0	0
*P*^-^*T*^-^	0	0	0	0.008	0	0
*P*^+^*T*^-^	0	0.047	0	0	0.156	0
*P*^-^*T*^+^	0	0	0	0	0	0.156

The elements of the diagonal correspond to the functionally invariant mutations, i.e. changes in the BR without changes in the dynamics. Rows represent the initial, columns the final dynamics. In tables 3 and 4 we summarize the effects of single mutations, whereas in table 5 and 6 accumulated mutations are presented, i.e. multiple conditions of the BR changed in a successive manner.

**Table 5:** Transition probabilities for accumulated mutations C1-C4

	***G***^**+**^	***G***^**-**^	***P***^**+**^***T***^**+**^	***P***^**-**^***T***^**-**^	***P***^**+**^***T***^**-**^	***P***^**-**^***T***^**+**^
**C1**						

*G*^+^	0.212	0	0.082	0.071	0	0
*G*^-^	0	0.235	0	0	0.010	0
*P*^+^*T*^+^	0.035	0	0.113	0.052	0	0
*P*^-^*T*^-^	0.030	0	0.052	0.082	0	0
*P*^+^*T*^-^	0	0.020	0	0	0.007	0
*P*^-^*T*^+^	0	0	0	0	0	0

**C2**						

*G*^+^	0.005	0	0.008	0.006	0	0
*G*^-^	0	0.822	0	0	0.018	0
*P*^+^*T*^+^	0.006	0	0.013	0.011	0	0
*P*^-^*T*^-^	0.008	0	0.014	0.018	0	0
*P*^+^*T*^-^	0	0.056	0	0	0.016	0
*P*^-^*T*^+^	0	0	0	0	0	0

**C3**						

*G*^+^	0.247	0	0.026	0	0	0
*G*^-^	0	0.222	0	0	0.050	0.043
*P*^+^*T*^+^	0.016	0	0.008	0	0	0
*P*^-^*T*^-^	0	0	0	0	0	0
*P*^+^*T*^-^	0	0.100	0	0	0.118	0
*P*^-^*T*^+^	0	0.086	0	0	0	0.086

**C4**						

*G*^+^	0.825	0	0.069	0	0	0
*G*^-^	0	0	0	0	0.005	0.005
*P*^+^*T*^+^	0.042	0	0.011	0	0	0
*P*^-^*T*^-^	0	0	0	0	0	0
*P^+^T*^-^	0	0.011	0	0	0.011	0
*P*^*-*^*T*^+^	0	0.011	0	0	0	0.011

**Table 6:** Transition probabilities for accumulated mutations I1-I4

	***G***^**+**^	***G***^-^	***P***^**+**^***T***^**+**^	***P***^-^***T***^-^	***P***^**+**^***T***^-^	***P***^-^***T***^**+**^
**I1**						

*G*^+^	0.171	0	0.114	0.086	0	0
*G*^-^	0	0.024	0	0	0	0
*P*^+^*T*^+^	0.033	0	0.229	0.098	0	0
*P*^-^*T*^-^	0.024	0	0.098	0.122	0	0
*P*^+^*T*^-^	0	0	0	0	0	0
*P*^-^*T*^+^	0	0	0	0	0	0

**I2**						

*G*^+^	0.088	0	0.048	0.040	0	0
*G*^-^	0	0.398	0	0.007	0.029	0.007
*P*^+^*T*^+^	0.016	0	0.088	0.040	0	0
*P*^-^*T*^-^	0.013	0.015	0.040	0.061	0	0
*P*^+^*T*^-^	0	0.058	0	0	0.037	0
*P*^-^*T*^+^	0	0.015	0	0	0	0.001

**I3**						

*G*^+^	0.024	0	0	0	0	0
*G*^-^	0	0.165	0	0	0.098	0.039
*P*^+^*T*^+^	0	0	0	0	0	0
*P*^-^*T*^-^	0	0	0	0	0	0
*P*^+^*T*^-^	0	0.196	0	0	0.353	0
*P*^-^*T*^+^	0	0.078	0	0	0	0.047

**I4**						

*G*^+^	0.401	0	0.033	0.017	0	0
*G*^-^	0	0.088	0	0	0.032	0.032
*P*^+^*T*^+^	0.037	0	0.035	0.005	0	0
*P*^-^*T*^-^	0.009	0	0.003	0.001	0	0
*P*^+^*T*^-^	0	0.064	0	0	0.088	0
*P*^-^*T*^+^	0	0.064	0	0	0	0.088

The sum of the diagonal elements determines the fraction of the mutations without impact on the dynamics. It provides a measure of the robustness against perturbations. As our data show, all FFLs are highly robust against single mutations, they show similar values above 90%. In other words, upon single mutations the FFLs display low sensitivity against mutation, hence a low evolvability ***ε***. However, there occurs a significant change in the degree of evolvability if the effect of accumulated mutations is studied. We quantify the evolvability $\varepsilon \left(\Gamma_{i}\right)$ of a given topology as:

(7) $$E\left(\Gamma_{k}\right)=1-\frac{\sum_{i}\Omega_{k}^{m}\left(\phi_{i}|\phi_{i}\right)}{\sum_{i}\Omega_{k}^{1}\left(\phi_{i}|\phi_{i}\right)},$$

where $\sum_{i}\omega_{k}^{m}\left(\phi_{i}|\phi_{i}\right)$ represents the robustness against accumulated and $\sum_{i}\Omega_{k}^{1}\left(\phi_{i}|\phi_{i}\right)$ represents the robustness against single mutations. Since $\sum_{i}\Omega_{k}^{1}\left(\phi_{i}|\phi_{i}\right)>\sum_{i}\Omega_{k}^{m}\left(\phi_{i}|\phi_{i}\right)$, we have $0\leq E\left(\Gamma_{k}\right)\leq 1$.

### Entropy, kurtosis and the likelihood of appearance of FFL motifs

The degree of homogeneity of the distribution of probabilities for the implementation of any function can be used to characterize the level of flexibility or specialization or, complementarily, the plasticity of a given motif Γ_*i*_. Different measures can be used to quantify the homogeneity of a distribution, namely kurtosis (see Results and Discussion) or entropy. Here we point out some more details on the calculation of the extreme cases and their kurtosis and how the Shannon entropy can be applied equally effective for this task. The expression for kurtosis can be written [30] as:

(8) $$K\left(\Gamma_{i}\right)=\frac{n\left(n+1\right)}{\left(n-1\right)\left(n-2\right)\left(n-3\right)}\overset{n}{\underset{j=1}{\sum }}{\left(\frac{P\left(\phi_{j}|\Gamma_{i}\right)-<P>}{\sigma }\right)}^{4}-\frac{3{\left(n-1\right)}^{2}}{\left(n-2\right)\left(n-3\right)}$$

where <*P *> is the mean value of the probability distribution and *σ *is the standard deviation.

Considering the extreme case where the most specialized motif Γ_*s *_only implements a single function *ϕ*_1_, i.e. *P*(*ϕ*_*j *_| Γ_*s*_) = 1 if *j *= 1 and 0 otherwise, expression (8) reduces to:

(9) $$K\left(\Gamma_{s}\right)=\frac{\frac{7}{10}\cdot n\left(n+1\right)\left(n-1\right)-3{\left(n-1\right)}^{2}}{\left(n-2\right)\left(n-3\right)}$$

On the other hand, the most flexible motif Γ_*f *_implements all possible functions with equal probability *P*(*ϕ*_*j *_| Γ_*f*_) = 1*/n*. Here expression (8) leads to a mathematical indetermination $\frac{0}{0}$, because *P*(*ϕ*_*j *_| Γ_*f*_) = <*P >*. This indetermination can be solved calculating the limit

(10) $$\underset{P\left(\phi_{j}|\Gamma_{f}\right)\to <P>}{\text{lim}}K\left(\Gamma_{f}\right).$$

Applying L'Hopital's rule [31], expression (10) reduces to:

(11) $$K\left(\Gamma_{f}\right)=\frac{\left(n+1\right)\left(n-1\right)-3{\left(n-1\right)}^{2}}{\left(n-2\right)\left(n-3\right)}$$

The calculated kurtosis values are *K*(Γ_*s*_) = 6 and *K*(*Γ*_*f*_) = -3.33, knowing that *n *= 6 (number of different possible functions).

Finally, we apply the Shannon entropy [32] to our data set to describe the qualitative differences in homogeneity of the motifs' probability distributions and develop a measure for functional specialization. It is defined as

(12) $$H\left(\Gamma_{i}\right)=-\overset{6}{\underset{j=1}{\sum }}P\left(\phi_{j}|\Gamma_{i}\right)log_{2}\left[P\left(\phi_{j}|\Gamma_{i}\right)\right]$$

Again, we will first consider the extreme cases Γ_*s *_and Γ_*f *_to determine the range of all possible values. We find for the most flexible case *P*(*ϕ*_*j *_| Γ_*f*_) = 1/6 an entropy of *H*(Γ_*f*_) = *log*_2_(6). For the most specialized case Γ_*s *_(*P*(*ϕ*_1 _| Γ_*s*_) = 1 and 0 otherwise) the associated entropy is *H*(Γ_*s*_) = 0. Any FFL motif will have entropy values residing within this range. When correlating the FFLs' entropy and their abundance in figure 4b we find that the most abundant motifs show intermediate values. This trend coincides with what has been found when applying kurtosis, where too, *C*1 and *I*1 show intermediate kurtosis values. They do neither exhibit high flexibility nor high specialization, indicating that a trade-off between both features is associated to the most abundant motifs. It can be understood in terms of adaptability or in other words the motifs' plasticity to explore the landscape of possible dynamics under mutational pressure.

## Authors' contributions

SW: study conception, research design, mathematical analysis, manuscript writing. RS: study conception, research design, manuscript writing. JM: study conception, research design, mathematical analysis, manuscript writing. All authors have read and approved the final version of the manuscript

## References

[B1] Kirschner M Gerhart J Evolvability Proc Natl Acad Sci USA 1998 95 8420 7 10.1073/pnas.95.15.8420 9671692 PMC33871

[B2] Wagner A Robustness and evolvability: a paradox resolved Proc R Soc London B 2008 275 91 100 10.1098/rspb.2007.1137 PMC2562401 17971325

[B3] Ancel LW Fontana W Plasticity, evolvability, and modularity in RNA J Exp Zool 2000 288 242 83 10.1002/1097-010X(20001015)288:3<242::AID-JEZ5>3.0.CO;2-O 11069142

[B4] Stadler B Stadler P Wagner G Fontana W The topology of the possible: formal spaces underlying patterns of evolutionary change J theor Biol 2001 213 241 274 10.1006/jtbi.2001.2423 11894994

[B5] Alon U Network motifs: theory and experimental approaches Nature Reviews Genetics 2007 8 450 61 10.1038/nrg2102 17510665

[B6] Milo R Shen-Orr S Itzkovitz S Kashtan N Chklovskii D Alon U Network motifs: simple building blocks of complex networks Science 2002 298 824 7 10.1126/science.298.5594.824 12399590

[B7] Shen-Orr SS Milo R Mangan S Alon U Network motifs in the transcriptional regulation network of Escherichia coli Nat Genet 2002 31 64 8 10.1038/ng881 11967538

[B8] Mangan S Zaslaver A Alon U The Coherent Feedforward Loop Serves as a Sign-sensitive Delay Element in Transcription Networks J Mol Biol 2003 334 197 204 10.1016/j.jmb.2003.09.049 14607112

[B9] Wall M Dunlop MJ Hlavacek W Multiple Functions of a Feed-Forward-Loop Gene Circuit JMB 2005 349 501 14 10.1016/j.jmb.2005.04.022 15890368

[B10] Ozbudak EM Thattai M Lim HN Shraiman BI Oudenaarden AV Multistability in the lactose utilization network of Escherichia coli Nature 2004 427 737 40 10.1038/nature02298 14973486

[B11] Alon U Surette MG Barkai N Leibler S Robustness in bacterial chemotaxis Nature 1999 397 168 71 10.1038/16483 9923680

[B12] Barkai N Leibler S Robustness in simple biochemical networks Nature 1997 387 855 7 10.1038/43072 9202124

[B13] Ishihara S Fujimoto K Shibata T Cross talking of network motifs in gene regulation that generates temporal pulses and spatial stripes Genes Cells 2005 10 1025 1038 10.1111/j.1365-2443.2005.00897.x 16236132

[B14] Cotterell J Sharpe J An atlas of gene regulatory networks reveals multiple three-gene mechanisms for interpreting morphogen gradients Mol Sys Biol 2010 6 425 438 10.1038/msb.2010.74 PMC3010108 21045819

[B15] Mangan S Alon U Structure and function of the feed-forward loop network motif Proc Natl Acad Sci USA 2003 100 11980 5 10.1073/pnas.2133841100 14530388 PMC218699

[B16] Kalir S Mangan S A coherent feed-forward loop with a SUM input function prolongs flagella expression in Escherichia coli Mol Syst Biol 2005 1 2005.0006 10.1038/msb4100010 PMC1681456 16729041

[B17] Draghi J Wagner GP Evolution of evolvability in a developmental model Evolution 2008 62 301 15 10.1111/j.1558-5646.2007.00303.x 18031304

[B18] Prill RJ Iglesias PA Levchenko A Dynamic properties of network motifs contribute to biological network organization PLoS Biol 2005 3 e343 10.1371/journal.pbio.0030343 16187794 PMC1239925

[B19] Ghosh B Karmakar R Bose I Noise characteristics of feed forward loops Phys Biol 2005 2 36 45 10.1088/1478-3967/2/1/005 16204855

[B20] Cordero OX Hogeweg P Feed-forward loop circuits as a side effect of genome evolution Mol Biol Evol 2006 23 1931 6 10.1093/molbev/msl060 16840361

[B21] Ingram PJ Stumpf MP Stark J Network motifs: structure does not determine function BMC Genomics 2006 7 108 10.1186/1471-2164-7-108 16677373 PMC1488845

[B22] Lynch M The evolution of genetic networks by nonadaptive processes Nat Rev Genet 2007 8 803 813 17878896 10.1038/nrg2192

[B23] Solé RV Valverde S Are network motifs the spandrels of cellular complexity? Trends Ecol Evol 2006 21 419 22 10.1016/j.tree.2006.05.013 16764967

[B24] Solé RV Valverde S Spontaneous emergence of modularity in cellular networks J R Soc Interface 2008 5 129 33 10.1098/rsif.2007.1108 17626003 PMC2605507

[B25] Mazurie A Bottani S Vergassola M An evolutionary and functional assessment of regulatory network motifs Genome Biol 2005 6 R35 10.1186/gb-2005-6-4-r35 15833122 PMC1088963

[B26] Macía J Widder S Solé RV Specialized or flexible feed-forward loop motifs: a question of topology BMC Syst Biol 2009 31 3 84 10.1186/1752-0509-3-84 PMC2749051 19719842

[B27] Mangan S Itzkovitz S Zaslaver A Alon U The Incoherent Feed-forward Loop Accelerates the Response-time of the gal System of Escherichia coli J Mol Biol 2006 356 1073 81 10.1016/j.jmb.2005.12.003 16406067

[B28] Conant GC Wagner A Convergent evolution of gene circuits Nat Genet 2003 34 264 266 10.1038/ng1181 12819781

[B29] Teichmann SA Babu MM Gene regulatory network growth by duplication Nat Genet 2004 36 492 496 10.1038/ng1340 15107850

[B30] Balanda KP MacGillivray HL Kurtosis: a critical review Am Statistn 1988 42 111 9 10.2307/2684482

[B31] Spivak M Calculus 1994 Cambridge University Press 201 11

[B32] Shannon CE A Mathematical Theory of Communication Bell System Technical Journal 1948 27 379 423

